# A Quantum Probability Approach to Improving Human–AI Decision Making

**DOI:** 10.3390/e27020152

**Published:** 2025-02-02

**Authors:** Scott Humr, Mustafa Canan, Mustafa Demir

**Affiliations:** 1 Department of Information Sciences, Naval Postgraduate School, Monterey, CA 93943, USA; mustafa.canan@nps.edu; 2 Applied Cognitive Ergonomics Laboratory, Texas A&M University, College Station, TX 77843, USA; amhd@tamu.edu

**Keywords:** artificial intelligence, decision making, quantum decision theory, human-in-the-loop, generative AI

## Abstract

Artificial intelligence is set to incorporate additional decision space that has traditionally been the purview of humans. However, AI systems that support decision making also entail the rationalization of AI outputs by humans. Yet, incongruencies between AI and human rationalization processes may introduce uncertainties in human decision making, which require new conceptualizations to improve the predictability of these interactions. The application of quantum probability theory (QPT) to human cognition is on the ascent and warrants potential consideration to human–AI decision making to improve these outcomes. This perspective paper explores how QPT may be applied to human–AI interactions and contributes by integrating these concepts into human-in-the-loop decision making. To capture this and offer a more comprehensive conceptualization, we use human-in-the-loop constructs to explicate how recent applications of QPT can ameliorate the models of interaction by providing a novel way to capture these behaviors. Followed by a summary of the challenges posed by human-in-the-loop systems, we discuss newer theories that advance models of the cognitive system by using quantum probability formalisms. We conclude by outlining areas of promising future research in human–AI decision making in which the proposed methods may apply.

## 1. Introduction

Artificial intelligence (AI) is introducing additional capabilities to support decision making in a variety of different contexts. Besides additional capabilities, AI can proactively alter the decision space and act on information. However, at the moment, AI informational outputs may not be well understood [1] and, therefore, can change the nature of human decision making. Informational outputs from AI are also different from traditional sources of information due to the opaque underpinnings of these technologies [2,3,4]. Additionally, humans anthropomorphizing AI system capabilities, such as adhering to social norms when interacting with these technologies [5], can produce unwarranted interactions with machines. For these reasons, researchers must understand how AI technologies can potentially and inadvertently affect decision-making processes.

Understanding how AI technologies impact human decision-making processes is not without warrant. Establishments like the military are undergoing significant transformations in technology and organization due to the development of AI [6]. Several well-known accidents involving advanced technologies have demonstrated the potential harmful side-effects of similar automated systems when combined with human response mechanisms. For example, tragic accidents, such as the Patriot battery fratricide in Iraq [7], the USS Vincennes incident [8,9,10], the accidental killing of civilians in Afghanistan [11], and the downing of two U.S. (United States) Army Blackhawk helicopters [12], demonstrate the potential consequences of misconstruing information from complex automated systems. A common thread through these tragic accidents is how automated technology influenced human decision making. In the aforementioned cases, information from automated technology was misconstrued or misinterpreted and, thus, over trusted by the humans who contributed to incorrect decision making [13]. Moreover, there is increasing concern that humans are being beholden to decisions at machine speeds instead of systems in support of human decision-making processes [14]. Therefore, understanding how AI impacts human decision-making processes is necessary for improving human–machine interaction research.

The structure of the paper is as follows. First, we begin by defining AI, its limitations, and how it is taking over additional decision space. Then, we review human-in-the-loop decision making. Next, we introduce quantum probability theory (QPT) for cognition and how it can improve outcomes by better modeling uncertainty and order effects within human–AI decision making. Lastly, we conclude by outlining future research.

## 2. What Is AI?

There are numerous definitions of AI throughout the literature. In this plethora of definitions, AI and algorithms are used interchangeably throughout the research. In its simplest description, an algorithm is a recipe or a set of instructions to accomplish a sequence of tasks or achieve a particular result [15]. Algorithms are often subcomponents of any AI system but may not be considered intelligent on their own. AI, on the other hand, is qualitatively different. In his seminal work that first coined the phrase “artificial intelligence” in 1955, John McCarthy [16] defines AI as “the science and engineering of making intelligent machines, especially intelligent computer programs” (p. 1). In Russell and Norvig’s [17] treatment on the matter, they define AI as “machines that can compute how to act effectively and safely in a wide variety of novel situations” (p. 19). The authors of [18] state that “AI is defined as the ability of a computer system to sense, reason, and respond to the environment” (p. 61). While these definitions provide a diverse characterization of AI, contemporary advances have pushed for additional divisions.

More recently, some researchers suggest that AI is characterized by the ability to imitate human information processing, while also alleviating cognitive workloads for humans [19,20]. Moreover, AI can also be portrayed as weak AI or strong AI [21]. Weak AI is characterized by programs that mimic human intelligence, such as classification, or that use natural language processing techniques, while strong AI is described as having a cognitive system that can reason and have goals similar to a human mind [22].

Different approaches to AI over the years have resulted in different kinds of performance. A rules-based approach, sometimes referred to as expert systems, has provided limited abilities. However, developing rules for every system edge case and verification proved too intractable for rules-based systems and, thus, found limited application [23]. Today, cutting-edge AI employs large-scale frontier models that are powering the generative AI (GenAI) revolution [24]. While these systems are proving highly capable in many areas, they are not without their challenges [25]. For these reasons, this paper will make use of a GenAI example to elucidate the current and forthcoming challenges to human–machine decision making.

In this paper, we define AI as a computer program that automates one of several aspects of human intelligence (e.g., categorization, classification, natural language processing, and decision making). This definition allows us to consider AI as an augmentation of a human decision-making process. We also recognize that such a narrow definition may not capture all aspects of what AI can currently be designed to perform, which is beyond the scope of this paper.

AI systems are created to accomplish a particular task commensurate with its design. However, most AI systems today are considered “brittle” [26] due to their training on constrained data sets or narrowly defined tasks. For instance, an AI designed for a classification task may not necessarily perform accurately at natural language processing. Yet, some AI programs today employ new techniques, as seen in Google’s Gato program, which is starting to break down this narrow barrier [27]. Notwithstanding a few instances of more versatile AI, most AI today remains limited.

### 2.1. Limitations of AI

AI can have several limitations and can promote unwarranted interactions. For instance, early research has shown that humans often anthropomorphize such intelligent agent-based systems by adhering to social norms when interacting with these technologies [5]. Automation errors on tasks that appear “easy” to a human operator can also severely degrade trust and reliance on automation [28]. Yet, AI has been characterized as ignorant and suffers from what some researchers identify as a Dunning–Kruger effect [29]. Similarly, these failures can be summarized by Moravec’s paradox, which highlights that AI can excel at high-level intelligence tasks, like chess and theorem proving, but struggles with simple tasks that require sensorimotor skills, such as stacking colored blocks or simple context-dependent social interactions, which are extremely simple for humans [30]. Particularly, recent strides in AI, driven by enhanced computational power and more sophisticated machine learning models, are beginning to address these challenges, yet tasks that are instinctive to humans continue to pose significant difficulties for machines [30]. Other research suggests that external AI traits (e.g., human-like appearance, human-like responses) can influence a human user’s mental models in unhelpful ways [31]. Advances to make AI decisions explainable have had mixed results. For instance, recent research has suggested that explainable AI does not necessarily improve user acceptance of AI advice [32]. For these reasons, it has become increasingly important for researchers and practitioners to understand how AI technologies are taking over additional decision space.

### 2.2. AI Aspirations: Taking over Additional Decision Space

AI is becoming more integrated into decision-making processes. This places humans in a position of responsibility and oversight for such AI advice. However, humans are increasingly disconnected spatially and temporally from what goes into algorithmic decision making [33], which makes it difficult to comprehend AI logic. This comprehension is essential in decision-making situations, which involve the rationalization of two more decision-making entities. Despite this, algorithms are gradually taking on traditional roles of management, such as assigning tasks to workers, worker compensation, and the evaluation of workers with little-to-no human oversight due to the sheer volume and scale of decisions [34]. While automated decision making may reduce human workload, it may also increase the uncertainty and predictability of outcomes. This is because the essence of decision making entails the choice amongst differing options [35]. More specifically, uncertainty can be characterized as strategic uncertainty. The authors of [36] define strategic uncertainty as “uncertainty about the actions of others in interactive situations” (p. 369). The actions of other agents (e.g., AI) may be difficult to understand or may unintentionally constrain decision options. For instance, if humans sense no degree of freedom in choosing amongst AI choice alternatives, then choice may be an illusion. In such cases, humans may begin to sense a degree of arbitrariness or sense randomness in decision making [37]. To understand these complexities, this next section embarks on explicating human-in-the-loop constructs.

## 3. Human-in-the-Loop Decision Making

AI is already supporting human decision making in many ways. From Netflix recommendations to Uber drivers being managed by sophisticated algorithms, AI is taking over more decision space traditionally performed by humans [38]. However, AI advancements are outpacing the conceptual coverage of human–computer decision making [39]. In some instances, AI is required due to the sheer scale or immediacy of the service, making human oversight in the decision process impractical [34]. Yet, many decisions still require human oversight, and keeping a human in the loop (HITL) is important [40]. For the immediate future, AI and humans will make contributions to automated decision-making processes [41]. Still, understanding the distinct roles humans can have within an HITL construct can provide additional insights into decision making with AI.

Humans can fulfill a variety of distinct roles when it comes to HITL constructs. The authors of [26] define an HITL as “an individual who is involved in a single, particular decision made in conjunction with an algorithm” (p. 12). In Table 1, the authors of [26] identified nine distinct roles a human may fulfill for an HITL system. These roles are not mutually exclusive and offer several improvements for HITL decision-making processes. While such roles can provide supervision to algorithmic decision making, a lack of comprehensible understanding in the dynamics of AI can affect the overall system and individual performance and inadvertently influence subsequent judgments and decision outcomes. Thus, systemic improvement to HITL decision-making processes with predictable methods requires the adoption of newer approaches to model human decision making that can capture and model decision uncertainty.

## 4. Quantum Probability Theory for Decision Making

The application of the mathematical axioms of quantum mechanics in decision modeling is breaking new ground in decision science. Despite the non-intuitive nature of applying the mathematical axioms of quantum mechanics to macro-level processes, the application of quantum formalisms to model human cognitive behaviors is gaining ascendency and broadening its application to new areas outside its original purpose [41,42,43,44,45,46,47,48,49,50,51]. For example, the concept of measurement/observation shares similarities to social anthropology by recognizing that observation in both fields changes the system [52]. In spite of these reasons, quantum formalisms require further explanation to clarify their benefits in modeling human behavior.

Applying quantum mathematical axioms to human decision making does not assume any “quantumness” about the system of interest or that the human brain exhibits a quantum process. As the authors of [53] state, “Quantum probability theory, as employed by behavioral scientists, simply concerns the basic probability rules of quantum theory without any of the physics” (p. 3). In terms of uncertainty, modeling decision making with quantum mathematical axioms operationalizes a crucial dynamic, that is, judgment *creates* rather than records what existed right before the judgment [emphasis added] [44]. From the uncertainty point of view, one advantage for operationalizing this dynamic means that decision models include an ontic type of uncertainty. We adopt the description of ontic uncertainty described in [47] as an internal state of uncertainty a person feels about providing certain responses (e.g., choices, judgment). Ontic uncertainty can emerge when humans attempt rationalizing from a cognitive state that is simultaneously comprised of a combination of many different states of possibility or in quantum parlance, a superposition (e.g., thinking about combinations of all potential perspectives) [54]. Put another way, ontic uncertainty describes the cognitive indeterminacy represented by a combination of all possible outcomes [44]. The crux of including this type of uncertainty in a decision model is that it is internal to the decision maker. The conventional modeling of human decision making under uncertainty has fallen short in capturing the ontic type of uncertainty because of the limitation of the classical probability theory (CPT) [55]. Therefore, the adoption of QPT principles can ameliorate human–AI decision making for the following CPT related limitations.

### 4.1. Cognitive Errors That Affect Decision Making

Daniel Kahneman’s work around cognitive biases is a good example to elucidate the modeling differences between CPT and QPT. His experiments and follow-on studies have repeatedly demonstrated the phenomenon of the anchoring effects of information [56,57]. Anchoring effects are introduced by decision makers taking into account an informational cue that can steer judgment towards an appropriate signal [58]. However, the developed explanations for anchoring effects either lack a rigorous mathematical formulation or rely on set theoretical constructs based on CPT (e.g., Bayesian models) [59]. Hence, such CPT approaches, such as Bayesian inference models, lack generalization and limit the use of theories to ad hoc descriptive explanations.

The line of decision modeling research based on QPT has also shown more tractable explanations for informational order effects over CPT. For instance, the authors of [44] analyzed a Gallup poll from 1997 that asked 1002 respondents, “Do you generally think Bill Clinton is honest and trustworthy?” and then asked the same question about Al Gore. Half of those respondents were instead asked the same question in the opposite order. The results demonstrated a 15-point percentage difference between the two conditions, which is not negligible. More importantly, QPT-based models can capture this difference, while CPT-based models fail to explain this difference. Similar effects can emerge in AI/ML-supported decision-making situations when influenced by the trust in AI as an information provider. Therefore, building and engineering decision support systems can significantly benefit from QPT-based models [48,60].

Due to the limitation of CPT, thus-far determined CPT violations are categorized fallacies, such as conjunction and disjunction fallacies [44,56], the Allais paradox [61], the and Ellsberg paradox [62]. These previous studies demonstrate the relevance of decision-making fallacies to HITL-AI systems’ decision-making environment [63,64]. For these reasons, addressing the order effects and the decision fallacies is critical for HITL-AI systems for decision making for several reasons. First, it is argued that taking a measurement or interacting with a system “creates rather than records a property of a system” (Busemeyer and Bruza, 2014 [44], p. 3). For instance, the authors of [63] demonstrated how categorization by a computer versus human categorization can give rise to violations of total probability. Other studies have also shown how events might become incompatible because of how related information is processed sequentially with the first piece of information setting a context for the second event (e.g., AB≠BA) [50,53,60,65]. Second, the incompatibility of events makes it impossible for a human to form a joint probability across decisions, which signals contextuality [41]. The inability to form a joint probability across decisions induces an ontic type of uncertainty. For these reasons, the order effects of information within HITL-AI systems are an important consideration in decision making.

### 4.2. Categorization–Decision

Another decision dynamic that poses challenges in AI-supported decision making is the categorization–decision paradigm. The categorization–decision lines of research demonstrated that, when a decision maker is asked to categorize and then decide vs. no explicit categorization and making a decision directly, participants consistently made violations of total probability. For instance, in a study conducted by [53,66], participants who first categorized a face (e.g., labeled them bad or good) had a higher probability of commensurate follow-on action (e.g., bad, then attack vs. good, then withdraw). However, when the categorization step was removed, participants reversed their behaviors; thus, total probability of the two-event outcome was violated [63]. Moreover, the authors of [63] demonstrated that computer-provided categorization–decision vs. direct decision results in total probability violation, which has been observed in various categorization research studies. This total probability violation alludes to how completing or overlapping signals in the information environment produce a disturbance in one’s judgment, or what is referred to as interference effects, when there is no categorization. In an online experiment conducted by [50], similar violations in total probability were observed in a categorization–decision task for an imagery analysis task supported by an AI assistant. For these reasons, AI-supported decision making highlights the need for more comprehensive modeling techniques to account for these human behaviors.

### 4.3. Interference Effects

Quantum models of cognition assume that evidence for decision making results from a cumulative process over time and can result in interference effects when multiple system measurements are taken [67]. For instance, the authors of [47] demonstrated interference effects when human choice behavior early in the process strongly affected outcomes exhibited in the mean preference ratings later in time over a no-choice condition. The critical finding of this line of research is that, when a participant is given the categorization (e.g., given by a computer), the total probability difference between the given categorization and direct decision conditions is still observed; interference effects, negative or positive, are captured via the total probability difference [63,64]. The examples cited challenge much of the conventional cognitive psychological research on human behavior that still capitalizes on the axioms on CPT to model human–AI decision making without a truly satisfying answer that accounts for empirical violations of rational behavior.

Developing and designing tomorrow’s decision support systems (DSS) augmented with AI must, however, account for quirks of the human mind that give rise to dynamics and subsequent interference effects in human decision making. The application of QPT to AI DSS will benefit from having a framework that can account for the cognitive consequences of processing incompatible information [60]. The application of QPT to HITL-AI systems can open the door for not only better modeling of these systems but to potentially engineer environments that improve decision making.

## 5. How Quantum Cognition Can Improve Human-in-the-AI-Loop Decision Making

QPT can help improve HITL-AI decision-making models by introducing the incompatibility condition that gives rise to interference effects. Interference effects can result in volatility in the outcome probabilities; hence, interference effects can give rise to erroneous understanding or the oversight of emergent behaviors in complex situations [68]. Therefore, conditions that can give rise to the incompatibility of different states must be accounted for in the models [69].

Improving the design of the HITL-AI systems with QPT-based models provides at least two additional benefits over CPT-based approaches: (1) a more comprehensive modeling of uncertainty and (2) a facility to investigate and determine the conditions that can give rise to incompatibility between decision variables. First, QPT can capture uncertainty more comprehensively (e.g., over evidence accumulation models), because it assumes that the cognitive system is not in a definite state [50]. In QPT, one’s cognitive state can be characterized as oscillating between possible states, also known as a superposition of states. However, once an interaction takes place, such as answering a question, the system, transiently, is put into a definite state. Using CPT-based models assumes that a cognitive system is at a definite state at any given time. Therefore, according to such classical models, a measurement records what existed before it takes place. As a result, CPT-based models fail to capture the cognitive vacillation that occurs in the mind of a subject [44], which is the internal uncertainty of the decision maker. Therefore, uncertainty cannot be accounted for in the decision model using CPT-based models. For example, uncertainty is synonymous with experiencing ambiguity (an indefinite state), which is self-evidently true; otherwise, if one knew what state they were in, they could easily inform themselves of this state [70]. In this case, the existence of the second perspective sets an implicit context that may give rise to interference effects. Modeling decision making with QPT allows modelers to investigate and determine compatibility relation among perspectives and associated pairs of variables. Second, QPT can model both epistemic and ontic types of uncertainty. Epistemic uncertainty is characterized by an observer’s lack of knowledge about the state of a system at a particular time, which can be resolved through acquiring additional knowledge [45,54]. Ontic uncertainty describes a person’s internal uncertainty regarding a specific response, such as a decision amongst different options [44,45,47]. The distinction between epistemic and ontic uncertainty is not recognized in cognitive models that use CPT-based models but should be considered in the design process of next the generation of HITL-AI systems for improved decisions.

### HITL-AI Examples

The proliferation of AI DSS to support human decision making continues to expand. AI, in general, is introducing a host of additional capabilities across the organizational spectrum [71]. Specifically, AI DSS is on the ascent in the medical field [72], industrial control [73], strategic decision making [74], and the military on a number of different fronts [75,76]. While the anticipated benefits of AI are many [77], there is also concern for a number of negative implications of AI supporting human decision making [78,79]. For instance, such AI systems are often opaque and can introduce uncertainty into human decision making [80]. For these reasons, it is important to show how several recent studies demonstrate how AI is able to influence human decision making.

HITL decision making supported by AI provides a fertile field for examining human–machine (i.e., AI) interactions. The HITL construct is particularly important, because it models the canonical observe, orient, decide, and act (OODA) loop introduced by Air Force colonel John Boyd [81]. When people discuss human and machine interactions that involve an HITL, they are often implicitly referring to the OODA loop, especially in military contexts [82]. In one such study by the authors of [50], the researchers found that AI-supported decision making provided both a bolstering effect and human behaviors that violated the concept of total probability. Moreover, this study was able to demonstrate the ability of quantum probability to better model human behaviors than CPT, such as Markov evidence accumulation models. Similarly, the authors of [48] found that a quantum model provided a better means to predict the evolving reliability ratings of an AI more than a Markov model. In another study, the authors of [83] demonstrated how applying QPT improved the understanding the decision-making process in human–machine interactions as an improvement over CPT. Consequently, exploring how QPT can ameliorate decision making with AI is an important step, as AI begins to augment the human decision landscape, particularly with the advent of large language models (LLMs).

Today, many search engines are powered by AI to leverage pattern detection and optimization to boost particular content [84]. AI technologies, such as LLMs (i.e., ChatGPT, Claude, Mistral AA, Google Search Labs), are now embedded in search engines as well [85]. In the case of search, a human directs a search engine to explore specific terms of interest and receives a ranked the output. To illustrate the possible contributions of QPT-based models, a general search engine story from The Cyber Effect [86] is discussed with a QPT-based model perspective.

“*Lisa went hiking with her friend during tick season; while hiking, she and her friend talked on assorted topics including ticks and Lyme disease. Upon returning home, she became worried about ticks. After examining her body, she finds a tick and removes it with the help of the information she obtained via online search. After removing the tick, she began searching about Lyme disease and its symptoms. She clicked from one search result to another, and after visiting various web pages, her anxiety increased. She continued reading more about Lyme disease. Click after click, she tumbled into medical webpages, only increasing her anxiety. Lisa lost track of time, often missing relevant information during her searches that might have been calming. Based on her frenetic searches, Lisa started to think that she had Lyme disease and ended up visiting an urgent care in the morning. The doctor confirmed that Lisa did not have Lyme disease but incurred costs for the unnecessary visit and contracted a virus from another patient who was visiting the same doctor’s office*”.

[86]

The similarity between categorization and decision for Lisa’s example is as follows. To decide Lyme positive, there are symptoms (e.g., having red eyes) that need to be categorized (explicitly). While searching online medical information about Lyme disease, Lisa could not make any categorization for the symptoms; for example, during her internet searches, Lisa observed her eyes had become red, the red eyes could be due to extended screen time while searching in the dark. Despite this context, red eyes were instead interpreted as a symptom of Lyme disease. Moreover, every new piece of AI-provided information introduces a new perspective for each introduced category and possibility. If perspectives are not categorized, they can continue to influence subsequent decision making.

**Figure 1:**
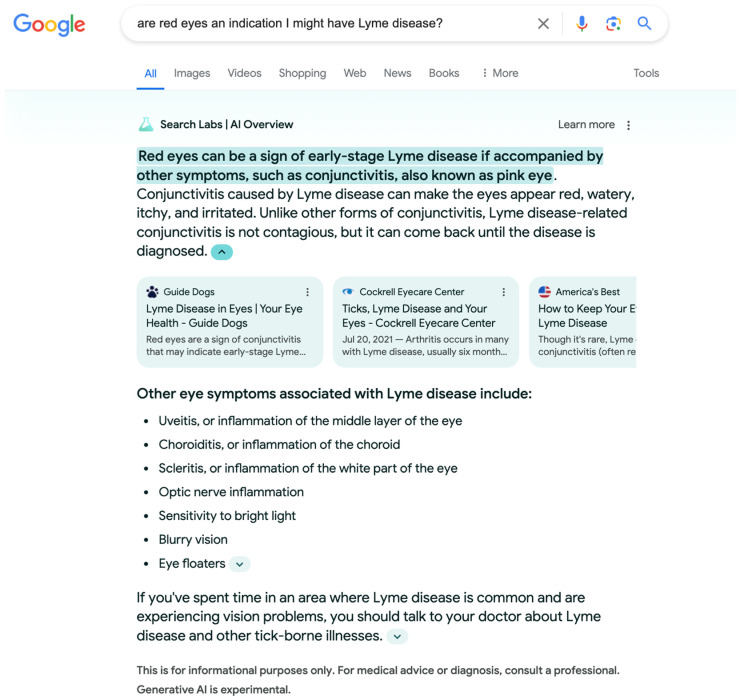
Google Generative AI experimental search engine output for a query on Lyme disease asked on 5 July 2024. Screenshot from Google Labs AI Generative AI experiment search result demonstrated through a number of informational cues, such as highlighting text, minimization of important text using a smaller font, and a list of conditions that a user can screen themselves against and can influence user behavior or increase their uncertainty, which may increase anxiety. Results are for informational purposes only.

One can argue that using more interactive advanced search engines may obviate this problem. However, as the authors of [87] articulate, language is indeterminate, and every phenomenon comes with presuppositions that are contextual and the enunciation of those presuppositions cannot guarantee a compatible perspective to process information. For instance, Figure 1 shows how a Google Generative AI experiment for searching, an advanced large language model (LLM) AI, can provide helpful advice but can result in vacillation, because users may not be familiar with the provided medical information. As a result, new perspectives become incompatible and begin influencing (forming context for) each other (e.g., other enumerated symptoms). Hence, the mental indecisiveness concerning the symptom categories can bolster positive Lyme disease belief. Since Lisa did not make any categorization while searching online, every new piece of information introduced new states to the cognitive system. If these new states do not match with perspectives of the information-processing individual, they begin influencing each other, which results in vacillation.

After visiting a doctor, Lisa would establish the definite category choices for the symptoms from a professional with supported test results. Thereafter, Lisa would resolve her indecisiveness concerning the symptoms and would have a negative belief about having Lyme disease (because symptoms are not related to Lyme disease). Lisa’s negative belief about her condition would, therefore, not be influenced by any mental indecisiveness concerning potential intermediate symptoms. Hence, her Lyme disease probabilistic outcomes would not have volatility. To consider potential indecisiveness in the design of HITL-AI systems through the lens of a similar interaction with an AI search engine, an AI can (1) push the human to a specific decision [88] and (2) generate uncertainty concerning the decision outcomes and bolster one of them.

**Figure 2:**
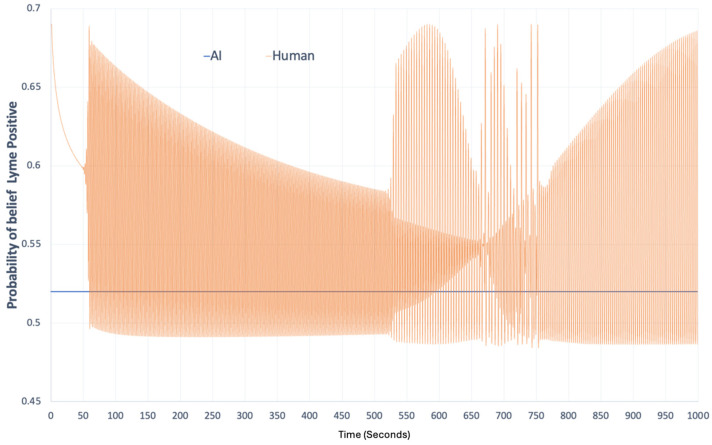
Lyme disease probability in the mind of Lisa over time. Temporal oscillation of probability of Lyme positive belief for Lisa; the initial probability of Lisa’s Lyme positive belief is 0.69. See Appendix A for additional explanation.

To demonstrate the second situation following the discussion in [83] that is based on quantum decision theory (QDT) and Kullback–Leibler relative information gain approach [89,90], a simulation for a two-decision outcome result was run with the following scenario. Suppose, based on the regional tick bite and Lyme diagnoses data, a search engine assumes that 52 percent of the people with tick bites contracted Lyme disease. Due to this data, the search engine optimizes its results in response to Lisa’s search entries in such a way that anyone reading the suggested pages would think that the probability of having Lyme disease is 0.52. As shown in Figure 2, a probability of positive Lyme disease belief for Lisa can oscillate in the case of continuous inquiry from a search engine. The oscillation shown in Figure 2 represents the ontic type of uncertainty that can bolster a specific decision outcome. We consider this oscillation to be the ontic type of uncertainty, because it is internal to the human; it is not directly accessible to an observer unless the human elicits her/his decision at any given time. This oscillatory behavior may converge later in time or diverge depending on Lisa’s cognitive schema. Therefore, capturing this type of uncertainty is important for properly modeling HITL-AI interactions in the design of decision support systems. Appendix A provides a detailed explanation of the mathematical formalisms for the proposed example.

These examples demonstrate how AI can potentially inadvertently generate uncertainty into human decision making. AI is already raising concerns in many circles from a number of organizations [91,92] to the military [79,93,94]. Recent collaborations between the developers of some of the most advanced LLMs and defense contractors [95,96] raise a number of questions about how humans will interact with these advanced AI decision aids. Moreover, national militaries will need to develop new ways of testing and benchmarks for the performance of these human–machine systems. These developments will create a host of new opportunities for interdisciplinary research programs to help address the complexity involved in fielding such systems at scale and in the many uncertain environments these systems will operate in. For these reasons, new methods, such as QPT, should be explored more rigorously in human decision making that leverages AI DSS.

## 6. Discussion

Design considerations that capitalize on QPT-based decision models to improve human and machine interactions are still in their infancy. Some research has suggested that the formalisms of QPT may be applied by machines for helping cognitively impaired humans (e.g., dementia or Alzheimer’s) achieve specific goals (e.g., washing hands, taking medications) [88]. While classical computers developed on Von Neumann architectures can calculate QPT, current work by [97,98] is demonstrating how quantum computers can also be leveraged as well for cognitive modeling. However, modeling human behavior using QPT to improve decision making in the moment could suffer from some practical constraints. For instance, developing real-time measures that can serve as surrogates for uncertainty are still being explored [48,88]. Yet, specialized chipsets and other hardware could be specially configured to optimize the speed and scale of such calculations. Nevertheless, such concerns must be considered if QPT will be used in high-stakes and high-tempo situations.

The same considerations can also apply to HITL-AI systems. Knowing the shortcomings of human reasoning and information processing, machines can better model human cognitive processes with QPT to account for how (1) humans are influenced by the order of information (e.g., humans should decide before AI reveals its own perspective; knowing when and whether to solicit for AI advice would lead to different decision outcomes [99]); (2) new pieces of information can result in incompatible perspectives and higher ontic uncertainty. Considerations for these design parameters could improve engineering AI systems for decision making through better predictability of human–machine interactions. Consequently, HITL-AI systems may be engineered to move human decision making towards a more Bayesian optimal choice [88]. With these design aspects in mind, much work still needs to be carried out.

## 7. Future Research

It is clear that humans and AI systems will increasingly engage in shared decision making. What decisions are ceded to AI is still a work in progress. Decision speed will also be an area of great concern for some areas of decision making (e.g., military, stock trading). How work practices are managed with the integration of human and machine technologies requires future research [100]. For instance, increasing pressure to continually shorten decision cycles is a reality in most militaries today, especially at the military tactical level for finding and engaging targets [6]. HITL-AI systems may accelerate such decision cycles. Future research will need to address how speed affects HITL-AI decision making in high-tempo and ethically significant operations and what can be done to improve outcomes.

The use of AI DSS can also raise a number of ethical concerns. From the medical field [101] to the military [102], AI DSS is providing both benefits and harms. New lines of research could explore how QPT can best model uncertainty and investigate what interventions could improve human decision making supported by AI DSS. Additionally, with AI prejudices becoming a major concern [103], QPT could provide intermediations that can alert users to properly considering alternatives that can help avoid automation bias.

Order effects have also been seen in human trust towards algorithms. For instance, Fenneman et al. (2021) [104] found that humans exposed to algorithmic managers first exhibited lower thresholds for trust but higher thresholds for trust when they were introduced to human fund managers first. Additional research is needed to help understand how oscillatory behavior might affect trust in human decision making supported by AI. It is also unclear from the current research how such oscillatory behavior could contribute to decision fatigue over extended interactions. Therefore, future research should address different interaction dynamics under multi-agent decision making (i.e., human and AI) that may improve trust interactions and other physiological indicators in HITL-AI systems.

Modeling human–machine interactions in real time should be the pursuit of future research. A great deal of modeling with QPT and CPT is a result of post hoc analysis and comparison. A logical next stage is to use current QPT and CPT models to create system interventions to investigate how both models compare to real-time decision making. Research [48,88] has proposed in situ measures that could help instrument such experiments. However, QPT models and CPT models alone may not provide the best approach. Recent research [47,50,105] has shown how QPT and CPT can be combined into a single hybrid function to model system behaviors called a quantum open systems approach. Therefore, new research that employs a hybrid approach could prove promising for modeling noisy and chaotic decision-making environments.

HITL-AI systems are anticipated to have second and third order effects on decision-making processes. It is projected that humans will continue to cede decision-making space to AI potentially without their awareness [99]. This can lead to several suboptimal outcomes and unanticipated effects. First, humans could become de-skilled in their own decision-making abilities. Such warnings of de-skilling have been raised in areas ranging from neurosurgery [106] and moral reasoning [107] to decision making itself [108] and could lower workers’ collective negotiating powers [109]. This zero-sum view is, however, not without warrant, as AI systems encompass additional decision space. These outcomes may also lead to difficulties in justifying additional human training as a back-up to AI systems due to being viewed as duplicative costs. These shortcoming will require additional research and experimental setups that can monitor and model both human and machine behaviors. Particular emphasis should be given to in situ field experimentation that captures real-world consequential decision making. As a result, future research should address these HITL-AI shortcomings, for it may de-skill workers, and what may be done instead to correct such outcomes.

The proliferation of AI systems also complexifies human oversight. For instance, it is anticipated that workers, such as radiologists, may become “orchestrators” of a plethora of AI-based systems and their concomitant workflows [99]. Research [110] suggests that algorithmic prescriptions may be overly complex, resulting in suboptimal decisions from the users’ inhibition to trust such systems. In other instances, humans providing oversight to AI systems may become “algorithmic brokers” to translate machine outputs to others [1]. Moreover, some scholars warn how “autonomous computer leaders” may make significant decisions without sufficient human oversight in the near future [39]. Researchers will also have to consider how to align experts and non-experts with AI systems. Research suggests that experts and novices can perceive AI outputs differently, which can result in different judgments and decision outcomes [99,111,112]. Future research and experimentation should, therefore, address both levels of expertise and leadership for HITL-AI systems.

## 8. Summary

In one of Simon and Newell’s [113] seminal papers, *Heuristic Problem Solving: The Next Advance in Operations Research*, they stated, “In dealing with the ill-structured problems of management we have not had the mathematical tools we have needed—we have not had ‘judgment mechanics’ to match quantum mechanics” (p. 6). This, however, is no longer the case. The application of QPT to decision making and similar efforts to formulate a concept of the quantum models of decision making [44] and QDT [89,90,114] have provided novel results that can better model uncertainty and human decision-making behaviors. Applying QPT to human and machine decision making is still at a nascent stage of development at the human–machine dyad level. Still, researchers must begin coming to grips with how AI can subtlety begin taking over this decision space and how researchers may help address this new phenomenon.

Developing or employing AI systems will always involve human decision-making processes. Adopting AI in any form will yield some element of human decision making, but researchers and practitioners will need to have a clear-eyed view of which elements are appropriate to delegate to machine intelligence [115]. Yet, it is still clear that machines such as AI cannot provide new affordances outside what it was trained to perform [116] or anticipate counterfactual outcomes [117]. These shortcomings should, however, not necessarily deter practitioners from adopting AI for improving decision making. Rather, such choices will affect how people may conceive HITL-AI as a complex sociotechnical system that requires an approach for joint optimization (e.g., structurally rational decision making, joint cognitive systems). Therefore, human response mechanisms to fulfilling roles within HITL-AI systems will likely evolve in diverse ways. To address these challenges, conceptual coverage must stay apace of AI advancements and should be closely monitored to ensure decision making is structurally rational and beneficial for all stakeholders.

## Figures and Tables

**Table 1:** Roles for humans in the loop.

Human Roles	Reason for Adding a Human in the Loop
1. Corrective Roles	Improve system performance, including error, situational, and bias correction
2. Reliance Roles	Act as a failure mode or alternatively stop the whole system from working under an emergency
3. Justificatory Roles	Increase the system’s legitimacy by providing reasoning for decisions
4. Dignitary Roles	Protect the dignity of the humans affected by the decision
5. Accountability Roles	Allocate liability or censure
6. Stand-in Roles	Act as proof that something has been carried out or stand in for other humans and human values
7. Friction Roles	Slow the pace of automated decision making
8. Warm Body Roles	Preserve human jobs
9. Interface Roles	Link the systems to human users

Adapted from [26].

## Data Availability

The original contributions presented in the study are included in the article, further inquiries can be directed to the corresponding author.

## Abbreviations

The following abbreviations are used in this manuscript:

AI	Artificial Intelligence
CPT	Classical Probability Theory
DSS	Decision Support System
GenAI	Generative Artificial Intelligence
HITL	Human-in-the-Loop
LLM	Large Language Model
QDT	Quantum Decision Theory
QPT	Quantum Probability Theory

## Appendix A

### Appendix A.1. Quantum Decision Theory and Relative Information Gain

In modeling interactions between a human and a search engine, the quantum decision theory (QDT) [118] is used for the following reasons. First, based on some concerns, humans begin with a search prompt, and a relative information gain begins. The human’s subsequent prompts are influenced/guided by this relative information gain. Second, based on initial and subsequent prompts, search engines develop a prospect and begin suggesting search results. These results are based on recent data in the area, search engine optimization, or other factors. The mathematical structure of QDT [118] captures some of the ontic uncertainty that the human experience by considering the optimized prospect of the search engine.

Suppose there are two decisions to choose: D_1_ and D_2_. According to QDT, the probability measure of choosing D_1_ for jth agent as a function of time can be expressed as:

(A1) $$p_{j}\left(D_{1},t\right)=f_{j}\left(D_{1},t\right)+q_{j}\left(D_{1},t\right)$$

where $f_{j}\left(D_{1}\right)$ is the utility factor representing the classical probability contribution, and $q_{j}(D_{1})$ is the interference term. The interference term is constrained by the quarter-law, and therefore, the non-informative priors for the average interference term are $q\left(D_{1,2}\right)=\frac{1}{4},-\frac{1}{4}$ [89,90,118,119,120]. The sign of the interference term depends on the decision being attractive or repulsive in the situation [89].

The utility factor, $f_{j}$, will be treated time-independently [90], and the quantum interference term depends on the information received by the human agent j, $M_{j}(t)$:

(A2) $$q_{j}\left(D_{n},t\right)=q_{0}\left(D_{n}\right)\exp\left(-\sum_{t=1}^{t^{\prime }}\varphi \left(t^{\prime },t\right)\mu_{j}\left(t\right)\right)$$

In Equation (A2), $\mu_{j}$ represents the information gained by a decision-making agent. In the case of two agents exchanging information, the information gain of the jth agent concerning the nth decision at time t can be expressed as [89,90]:

(A3) $$\mu_{j}(t)=\sum_{n=1}^{N}p_{j}\left(D_{n},t\right)\ln\frac{p_{j}\left(D_{n},t\right)}{p_{i}\left(D_{n},t\right)}$$

In the case of having two alternative decisions, the information gain function shown in Equation (A3) becomes

(A4) $$\mu_{j}(t)=p_{j}\left(D_{1},t\right)\ln\frac{p_{j}\left(D_{1},t\right)}{p_{i}\left(D_{1},t\right)}+p_{j}\left(D_{2},t\right)\ln\frac{p_{j}\left(D_{2},t\right)}{p_{i}\left(D_{2},t\right)}$$

Then, by using $p_{j}\left(D_{1},t\right)+p_{j}\left(D_{2},t\right)=1$, one can rewrite Equation (A4) as:

(A5) $$\mu_{j}(t)=p_{j}\left(t\right)\ln\frac{p_{j}\left(t\right)}{p_{i}\left(t\right)}+\left[1-p_{j}\left(t\right)\right]\ln\frac{1-p_{j}\left(t\right)}{1-p_{i}\left(t\right)}$$

### Appendix A.2. Relative Information Gain Between Agents

Before the internet era, Lisa would have gone to a doctor if she did not feel well. During the visit, the doctor would ask questions concerning her health; doctors have the training to make diagnoses and ask questions in a non-directive way [86]. In contrast, a medical webpage is designed to provide information to the consumers but not make diagnoses. As it occurred in Lisa’s situation, due to the increased anxiety, the terms that are entered into the search engine would become more Lyme-related; thus, the search engine would continue to recommend content that contains Lyme disease information. Consequently, the search engine’s prospect is to give more information, and the change in the information gain is negligible or takes longer than a human to change the recommendations, compared to the human’s information gain. Therefore, the prospect, utility factor f, for search engine is assumed to be constant.

In modeling Lisa’s situation as an interaction, first, we assume that humans interact with two types of machines. This includes the search engines she used and the web pages that are recommended and clicked on by her. Due to the nature of the interaction and rising anxiety, in our model, the information provided by the visited webpage at time t has a higher weight in Lisa’s memory than the earlier ones. Hence, the total received information by Lisa in the exponential power term in Equation (A2) becomes:

(A6) $$M_{Lisa}\left(t\right)=\sum_{t=1}^{t^{\prime }}\mu_{Lisa}\left(t\right)\cdot t$$

In QDT, when two human agents exchange information, Equation (A1), the probability of choosing decision D_1_ is

(A7) $$p_{j}\left(t+\tau \right)=f_{j}+q_{j}\left(t\right)$$

where the time step, τ, in Equation (A7), is 1 s in the model.

In the case of a human and search engine interaction, the model’s assumptions are as follows. Lisa’s utility factor, $f_{j}$, is time-independent, and the interference factor, $q_{j}$, is time-dependent; therefore, the probability equation is still time-dependent. Since search engines are optimized to find the most relevant web pages that can provide more information relevant to the terms that are searched, we suppose that there is no memory contribution for the inanimate agent, the interference term is zero, $q_{j}=0$, and the utility factor is time-independent.

To express the content of a medical webpage in terms of probability measures, the model’s assumptions are as follows. First, we assume that if a textual/semantic analysis of a medical webpage is conducted, a probability value can be assigned to the Lyme positive or negative; for example, probability of Lyme positive 0.52 means that the content of this webpage conveys information such that after reading the web content, the reader would think that he/she was 52% Lyme positive.

The initial value of the interference term is $q_{j}=0.20$ for two reasons. First, a positive value is chosen, because Lisa thought that she had Lyme disease and began searching for information about Lyme disease by using a search engine; therefore, the positive value indicates that she was attracted to any Lyme disease information. Second, due to the quarter limit [90,91,119,120] to avoid a very strong attraction, q = 0.20 is chosen.
